# Supplementary material for: Vertical Sleeve Gastrectomy Offers Protection against Disturbed Flow-Induced Atherosclerosis in High-Fat Diet-Fed Mice
Source: Int J Mol Sci. 2023 Mar 16;24(6):5669. doi: 10.3390/ijms24065669 (PMC10051344; doi:10.3390/ijms24065669)
Supplement: Supplementary file 1 [file ijms-24-05669-s001.zip › ijms-2256232-Supplemental data.pdf]

Supplemental data:

Supplementary Figure S1. Food intake, body weight and effects of SG on glucose metabolism in HFD-fed mice.

Food intake, body weight, and changes of glucose were assessed. Food intake and IPGTT curves were examined with *p*-value for trend and one-way ANOVA analysis with Tukey and Scheffee post-hoc tests. The differences in food intake in mice under chow or HFD were not significant. However, the HFD-fed mice had a minimal food intake after SG and resumed gradually (A). The difference of body weight in mice under HFD receiving SG and restrictive HFD was not statistically significant (B). The difference in IPGTT in HFD-fed mice that underwent SG was significant compared with the HFD-fed mice without SG. The IPGTT presented early peaking of sugar and turned to baseline gradually in HFD-fed mice underwent SG which indicated the insulin sensitivity improvement after SG (C). Compare to the baseline, paired *t* tests were performed for fasting blood glucose levels, insulin levels, and HOMA-IR at 0, 2, and 8 weeks for insulin resistance (D, E and F). After 2 weeks of HFD, hyperglycemia and HOMA-IR were induced, however, insulin levels were not significantly elevated. At 8 weeks, the levels of fasting blood glucose, insulin, and HOMA-IR were all lower in HFD-fed mice that underwent SG than that of HFD-fed mice that underwent sham operation for SG. However, fasting blood glucose, insulin, and HOMA-IR levels were not significantly

different after 2 and 8 weeks in chow diet-fed mice. Significant differences were not observed in the above-mentioned groups between undergoing carotid artery ligation or not.

Diet restriction was provided to HFD-fed mice intention to reduce the caloric intake and reduce the mice body weight (n=5-6 per group). We aimed to differentiate the benefits of SG was beyond sedulous starvation induced body weight reduction.

Chow diet: Picolab Rodent Diet 20; 4 kcal/g, 2% cholesterol, 5.7% fat; High fat diet: Teklad diet TD 88137; 4.5 kcal/g, 0.2% cholesterol, 21.2% fat; Harlan Tackle Co. Restrictive Diet (70% HFD mixed with 30% wheat bran)

* High-fat diet (HFD) compared with chow diet (CD) in mice. $^{*}p<0.05$, $^{**}p<0.005$, †Mice that underwent SG compared to those that did not undergo SG. $^{\dagger}p<0.05$, $^{\dagger\dagger}p<0.005$. The *p*-value for trend and one-way ANOVA were estimated for diet intake and serial changes of glucose in IPGTT with Scheffee and Tukey post hoc tests.

Supplementary Figure S2. SG attenuated HFD than restrictive HFD induced intimal thickening in carotid artery ligation.

H&E and Verhoeff-Van Gieson (VVG) staining with morphometric analysis for intima, media area, intima/media area ratio and elastin fragmentation in different diet groups were performed (A). The H&E and Verhoeff-Van Gieson (VVG) staining revealed significant intima and media hyperplasia in the left ligated carotid artery in HFD-fed mice than 70% restrictive HFD-fed mice that underwent carotid ligation and sham operation for SG (B-D). (n=4-6 per group). Similarly, the study showed SG significantly reduced intimal hyperplasia than restrictive diet in carotid ligation as well which indicated the effects on atherosclerosis by SG was beyond the body weight reduction.

Two-way analysis of variance was performed with one treatment parameter being normal chow, HFD, and HFD with SG, restrictive HFD and the other parameter being carotid ligation/no carotid ligation. A p-value <0.05 was considered to be statistically significant. **Any diet (HFD or restrictive HFD) compared with chow diet (CD) in mice with carotid ligation, * p<0.05, $^{**}p<0.005$, ††Mice with SG compared with sham operation of SG with carotid ligation. †p<0.05, $^{\dagger\dagger}p<0.005$.

Supplementary Figure S3. SG ameliorated HFD induced inflammatory reaction macrophage infiltration and MMP-9 upregulation in the ligated carotid artery than restrictive HFD. Representative light micrographs from the ligated left common carotid artery of the mice. Carotid artery immunostaining revealed ox-LDL lesions, macrophage infiltration and MMP-9 expression. The lower panels are the quantified immunohistochemical results by densitometry analysis (B, C and D). Scale bars: 100 µm. (n=6 per group)

Likewise, the study showed SG significantly reduced HFD induced inflammation, macrophage infiltration and MMP-9 upregulation than restrictive diet did in carotid ligation which marked again the effects on vascular inflammation and repair by SG was beyond the body weight reduction.

Two-way analysis of variance was also performed with one treatment parameter being normal chow, HFD, and HFD with SG, and the other parameter being carotid ligation/no carotid ligation. A *p*-value <0.05 was considered to be statistically significant. $^{*}p<0.05$, $^{**}p<0.005$, **Any diet (HFD or restrictive HFD) compared with chow diet (CD) in mice with carotid ligation, * p<0.05, $^{**}p<0.005$, ††Mice under HFD or restrictive HFD compared with Mice under HFD and SG under carotid ligation. †p<0.05, $^{††}p<0.005$.†Mice with SG with or without carotid ligation, $^{†}P<0.05$, $^{††}P<0.005$.

Supplementary figure S4. Immunohistochemical staining for ox-LDL and MMP-9 in the carotid artery of restrictive HFD-fed mice. Representative light micrographs from the ligated left common carotid artery of the mice. Carotid artery immunostaining revealed ox-LDL lesions and MMP-9 expression (A-D). (n=4-6 per group).

Two-way analysis of variance was performed with one treatment parameter being normal chow, HFD, and HFD with SG, and the other parameter being carotid ligation/no carotid ligation. A *p*-value <0.05 was considered to be statistically significant. **High-fat diet (HFD) compared with chow diet (CD) with carotid ligation, $^{**}P<0.005$, †† Mice with SG compared with non-SG mice with carotid ligation and HFD. $^{\dagger}P<0.05$, $^{\dagger\dagger}P<0.005$. $^{\#\#}$Mice under carotid ligation compared with mice under sham operation of carotid ligation. $^{\#}P<0.05$, $^{\#\#}P<0.005$
