# Supplementary figures and images for: Vertical Sleeve Gastrectomy Offers Protection against Disturbed Flow-Induced Atherosclerosis in High-Fat Diet-Fed Mice
Source: Int J Mol Sci. 2023 Mar 16;24(6):5669. doi: 10.3390/ijms24065669 (PMC10051344; doi:10.3390/ijms24065669)

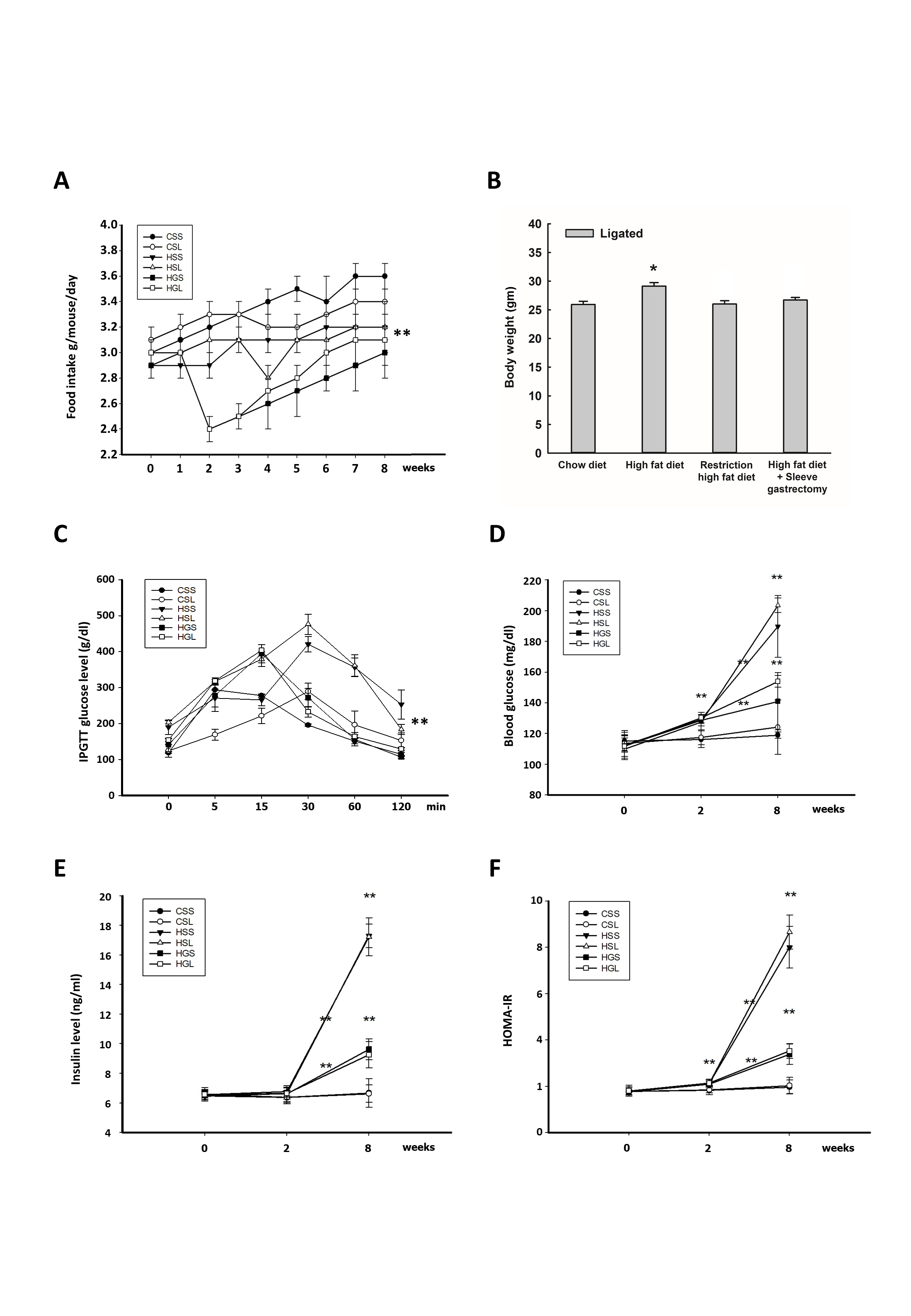

Supplement: Supplementary file 1 [file ijms-24-05669-s001.zip › Supplemental Figure S1.TIF]

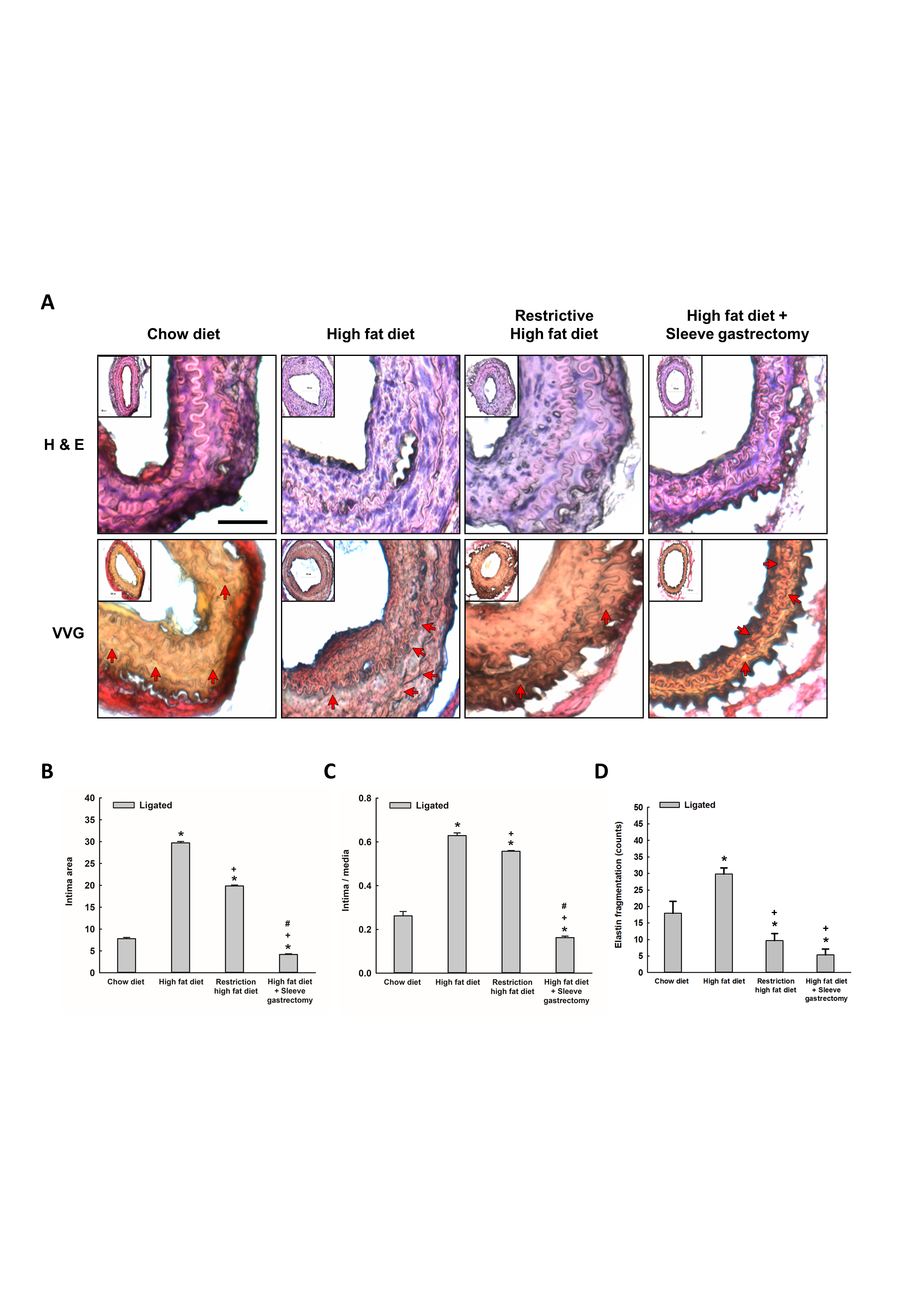

Supplement: Supplementary file 1 [file ijms-24-05669-s001.zip › Supplemental Figure S2.TIF]

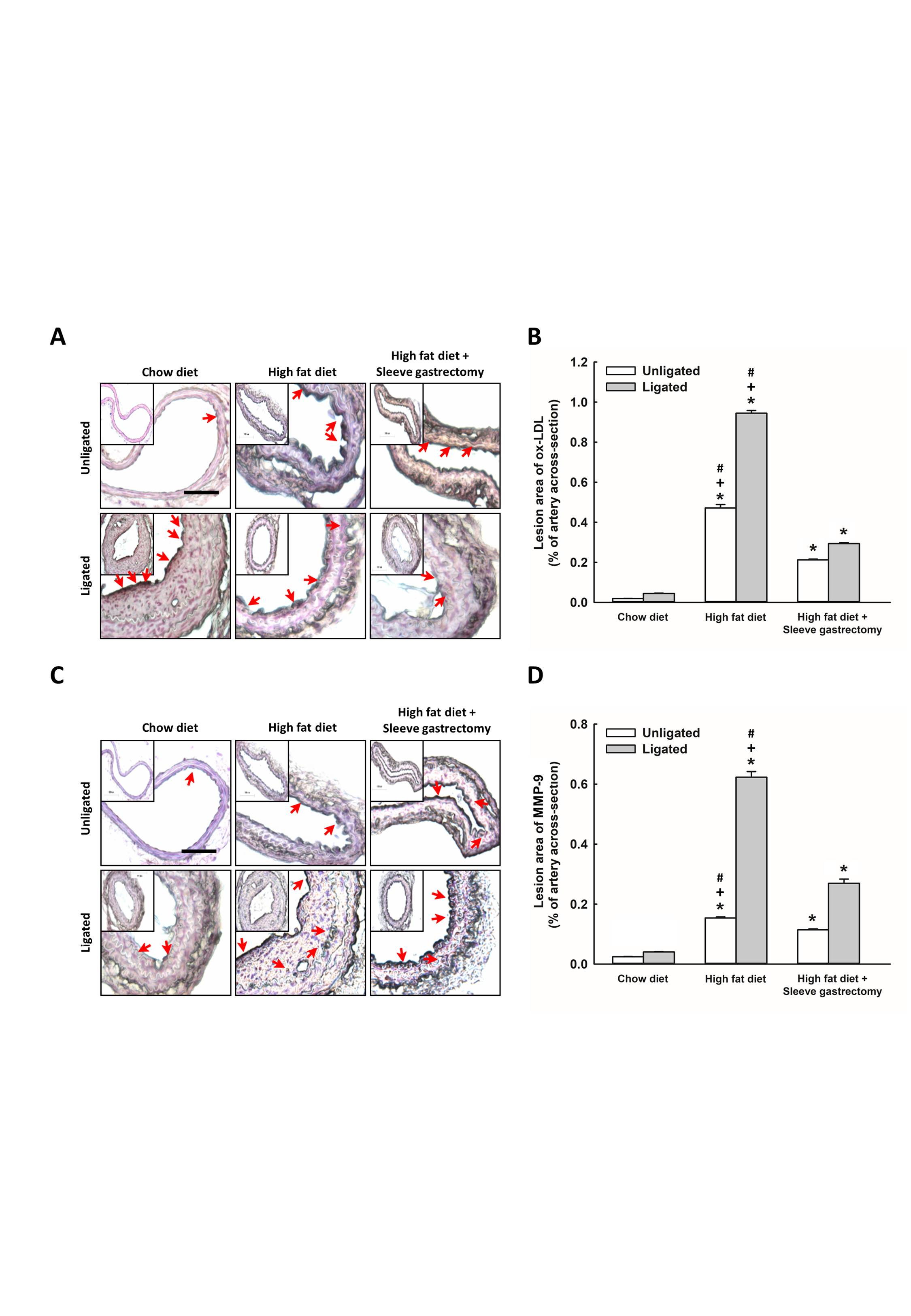

Supplement: Supplementary file 1 [file ijms-24-05669-s001.zip › Supplemental Figure S3.TIF]

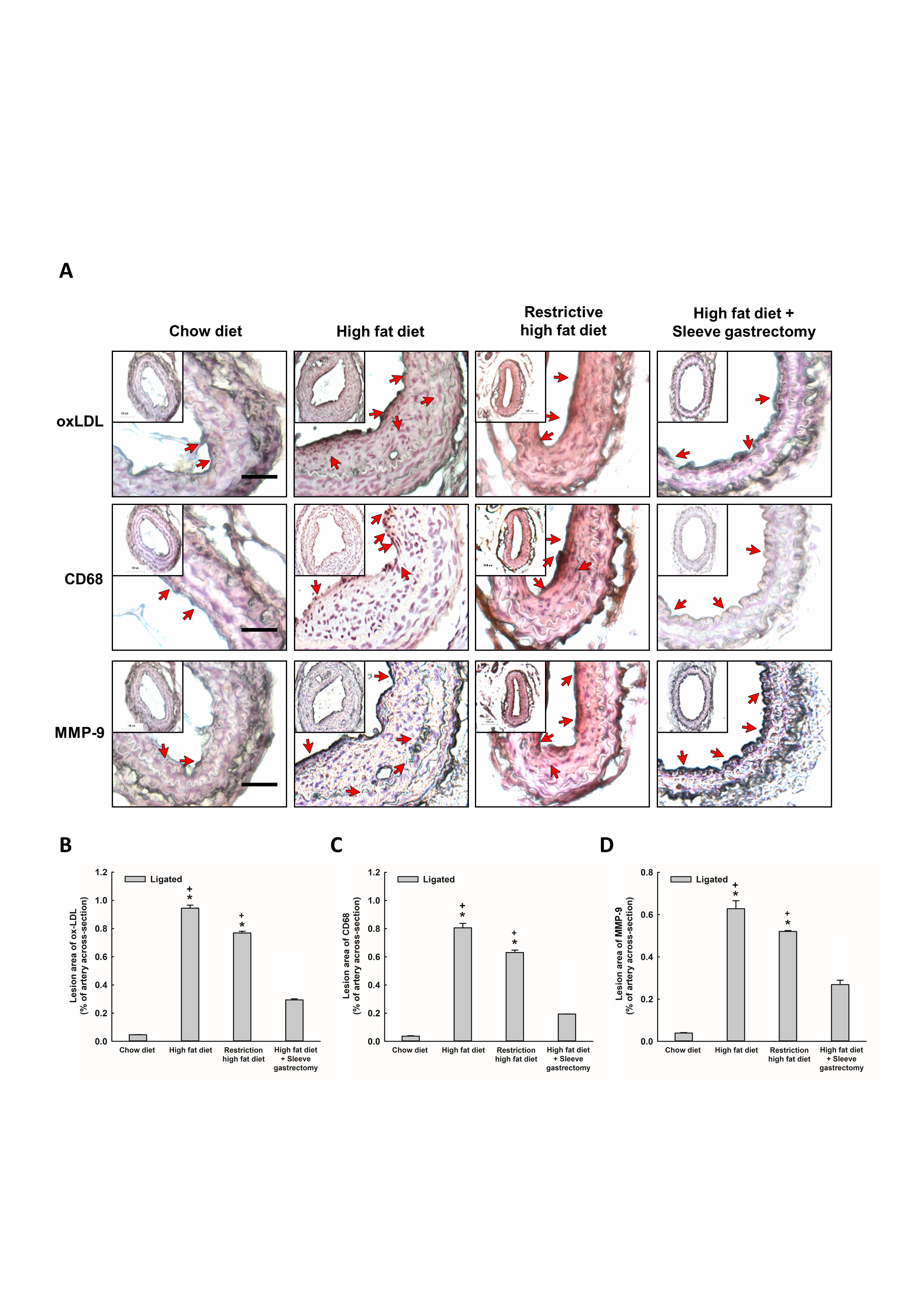

Supplement: Supplementary file 1 [file ijms-24-05669-s001.zip › Supplemental Figure S4.TIF]
